# Increasing Genomic Literacy Through National Genomic Projects

**DOI:** 10.3389/fgene.2021.693253

**Published:** 2021-08-12

**Authors:** Ana Nyasha Zimani, Borut Peterlin, Anja Kovanda

**Affiliations:** Clinical Institute of Genomic Medicine, University Medical Centre Ljubljana, Ljubljana, Slovenia

**Keywords:** genomic education, national genomic projects, personalized medicine, genomic medicine, patients, healthcare professionals, public, genomic literacy

## Abstract

Genomics is an advancing field of medicine, science, ethics, and legislation. Keeping up to date with this challenging discipline requires continuous education and exchange of knowledge between many target groups. Specific challenges in genomic education include tailoring complex topics to diverse audiences ranging from the general public and patients to highly educated professionals. National genomic projects face many of the same challenges and thus offer many opportunities to highlight common educational strategies for improving genomic literacy. We have reviewed 41 current national genomic projects and have identified 16 projects specifically describing their approach to genomic education. The following target groups were included in the educational efforts: the general public (nine projects), patients (six projects), and genomic professionals (16 projects), reflecting the general overall aims of the projects such as determining normal and pathological genomic variation, improving infrastructure, and facilitating personalized medicine. The national genomic projects aim to increase genomic literacy through supplementing existing national education in genomics as well as independent measures specifically tailored to each target group, such as training events, research collaboration, and online resources for healthcare professionals, patients, and patient organizations. This review provides the current state of educational activities within national genomic projects for different target groups and identifies good practices that could contribute to patient empowerment, public engagement, proficient healthcare professionals, and lend support to personalized medicine.

## Introduction

The field of genetics has, in the last few decades, provided an ever-increasing amount of tools to improve the health of individuals. At the same time, being a fast-advancing field of medicine, genetics has faced a continuous need to keep target groups adequately informed in order to enable them to access state-of-the-art health care. With the vast scientific advances of the last 20 years, such as the next generation sequencing, that have made sequencing of the whole genome accessible to the general public, the complexity of what is possible to determine, predict, prevent, and/or cure in human health has increased exponentially. Consequently, genomic, rather than genetic, literacy is now needed.

Genomic literacy is defined as “the capacity to obtain, process, understand, and use genomic information for health-related decision-making” (National Research Council, 1996; Institute of Medicine (US) Committee on Health Literacy, 2004; Hurle et al., 2013; Whitley et al., 2020), and, for this, a basic understanding of biology, inheritance as the etiology of hereditary diseases, and the concept of personal data management is needed (Dar-Nimrod and Heine, 2011; Stern and Kampourakis, 2017; Jackson et al., 2018; Hulsen et al., 2019; Youssef et al., 2019). These objectively complex topics need to be adjusted to the level of understanding of the target group before education can begin. For example, topics, such as personalized medicine of complex disorders and the genomics of rare diseases, need to be tailored in different ways for physicians and patients (Bennett et al., 2017; Goetz and Schork, 2018; Stoller, 2018; Ferreira, 2019; Ramalle-Gómara et al., 2020). Similarly, ethical, social, and legal aspects of genomics may be adjusted to different audiences (Taneri, 2011; Callier et al., 2016; Hartman et al., 2020; Wolf et al., 2020).

Genomic literacy is increasingly important due to the growing popularity of direct-to-consumer tests, an implementation of genomic medicine in routine healthcare and the necessity of public support for genomic research and is a prerequisite for an efficient implementation of personalized medicine, which has so far already changed the diagnostics and treatment of patients with rare diseases and cancer (World Health Organization, 2002; Dressler et al., 2014; ACMG Board of Directors, 2016; Brittain et al., 2017).

Currently, many governmental, non-governmental, and international organizations contribute to genomic education, through measures such as the incorporation of genomic topics into formal education, funding of educational institutions, the incorporation and establishment of training programs for genomic professionals, and through providing online provisions for the public and professionals and addressing the public through media engagement, etc. (Bennett et al., 2017; Goetz and Schork, 2018; Hyland and Dasgupta, 2019; Sabatello et al., 2019; Whitley et al., 2020).

To speed up the process of implementing personalized medicine, individual countries have established national genomic projects with various goals as previously reviewed (Kovanda et al., 2021). In order to achieve the main aims of genomic projects, such as determining normal and pathological genomic variation, improving infrastructure and finally implementing personalized medicine, it is crucial to increase genomic literacy among the public, patients, and professionals on relevant scientific and ethical issues (Bennett et al., 2017; Nakamura et al., 2017; Goetz and Schork, 2018; Ha et al., 2018; Hyland and Dasgupta, 2019; Sabatello et al., 2019; Wright et al., 2019; Whitley et al., 2020).

Therefore, national genomic projects, by addressing their major goal of promoting personalized medicine, substantially help genomics to enter into public awareness and thereby aid existing educational infrastructures in achieving genomic literacy.

Our goal was to review how education and promoting genomic literacy are addressed by the currently on-going national genomic projects, how these measures supplement existing genomic education, and to identify good practices of how they have been tailored to the key target groups of the projects educational outreach.

## Results

The 41 currently active national genomic projects were identified and analyzed through a systematic on-line search as previously described (Kovanda et al., 2021). As reviewed previously, the currently active national projects are very diverse and reflect the needs and resources of individual countries (Kovanda et al., 2021). The main aims of these projects are determining normal genomic variation (90%), pathological genomic variation (71%), improving infrastructure (59%), and achieving personalized medicine (37%). A total of 16 projects (39%, 16/41) specifically declared education as one of their aims (Table 1).

**Table 1:** Webpage resources of national genomic projects.

Country	Project names	Webpages/resources
Armenia	The Armenian Genome Project	http://armeniangenome.am/
Australia	Australian Genomics	https://www.australiangenomics.org.au/, https://www.genomicsinfo.org.au/
	Rare Cancers Australia – Your Cancer Journey	https://www.rarecancers.org.au/page/66/your-cancer-journey
Brazil	Brazilian Initiative on Precision Medicine BIPMed	http://bipmed.github.io/
Canada	Génome Québec – Education Platform Génome Québec Éducation et formations	http://www.genomequebec-education-formations.com/education-en
	Genome Canada	https://www.genomecanada.ca/
Cyprus	Center of Excellence in Biobanking and Biomedical Research	https://biobank.cy/
Finland	Finland’s Genome Strategy Working Group Proposal	https://julkaisut.valtioneuvosto.fi/bitstream/handle/10024/74712/URN_ISBN_978-952-00-3598-3.pdf?sequence=1&isAllowed=y
France	Plan France Médecine Génomique 2025	https://pfmg2025.aviesan.fr
Japan	Platform Program for Promotion of Genome Medicine | Japan Agency for Medical Research and Development	https://www.amed.go.jp/en/program/list/14/01/001.html
New Zealand	Genomics Aotearoa	https://www.genomics-aotearoa.org.nz, https://github.com/GenomicsAotearoa
Poland	Genomic map of Poland	http://www.ecbig.pl/page/genomic-map-of-poland/
Qatar	Qatar Genome Programme	https://qatargenome.org.qa/node/5
Saudi Arabia	Saudi Human Genome Program	https://shgp.kacst.edu.sa/index.en.html#program-objectives
Slovenia	Slovenian Genome Project	http://genom.si/
Switzerland	SPHN – Swiss Personalised Health Network	https://sphn.ch/
United Kingdom	Genomics England and Genomics Education Programme	https://www.genomicsengland.co.uk/ https://www.genomicseducation.hee.nhs.uk/
Uruguay	Urugenomes	http://urugenomes.org/en/the-project/

**Figure 1:**
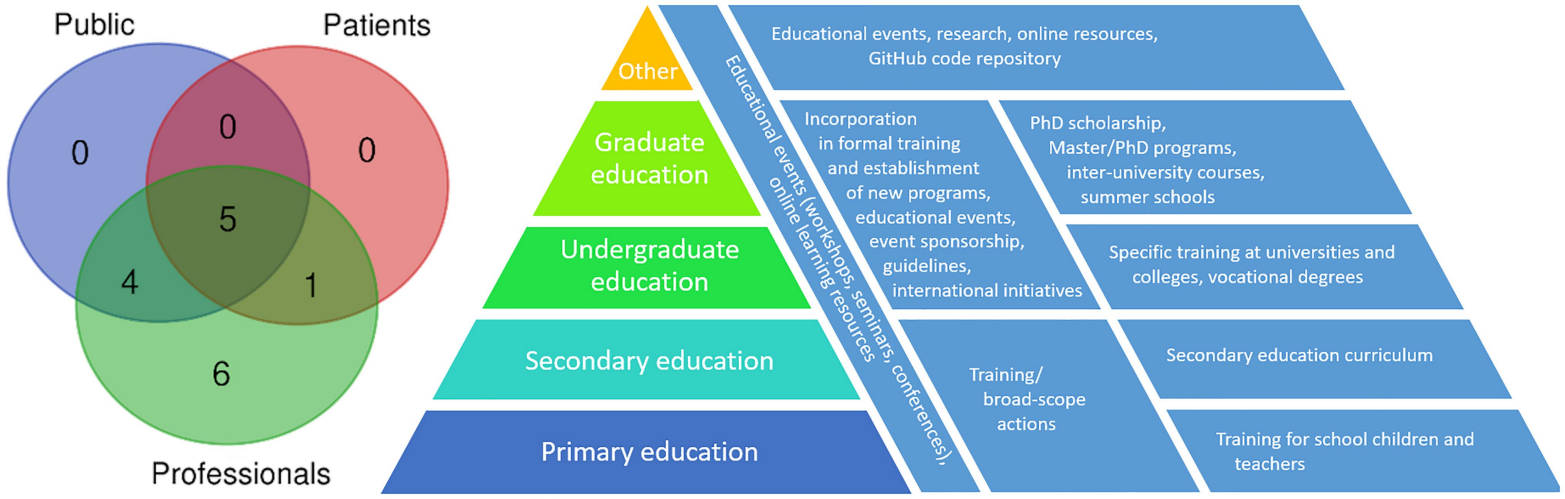
Overlap between the target groups of 16 national genomic projects and educational solutions and resources according to the level of education.

The projects with educational aims had three primary target groups; the general public (nine projects), patients (six projects), and professionals involved in genomics (all 16 projects; Figure 1), reflecting the projects’ general aims (Kovanda et al., 2021).

The various solutions addressing the many challenges of genomic education reflect the specific project aims and will be further discussed below. In addition to classic approaches, such as workshops and other educational events, there is a clear trend toward the utilization of online educational resources. The various educational resources are presented here according to the target groups and respective educational resources (Figure 1).

### The General Public

The public represents the most diverse group of stakeholders and includes individuals of all ages, education levels, professions, religions, and ethnicity. Nine of the national genomic projects specifically stated their efforts toward increasing the genomic literacy of the public.

The most common approaches among the projects targeting the general public include educational events, online platforms, and media and social media engagement (Table 1; Figure 1). Australia and the United Kingdom stated provisions for community engagement. Finland plans to empower its citizens to make informed decisions about genetic testing and study participation with the implementation of guidelines on the use of genomic data and online platforms, such as an educational genome portal, online genome tools, and virtual health services, which would enable users to interact with and use their genomic information. France, the United Kingdom, Canada, and Finland have begun to integrate genomic education into primary and secondary education by updating school curricula, providing teachers with specialized training or utilizing online educational platforms (LaRue et al., 2018; Whitley et al., 2020).

#### Primary Education Level

Primary education level is important and in many ways (simplification, emphasis on fundamental principles, and interesting examples of benefit to patients) similar to genomic education of the general public. For example, projects by the United Kingdom and France have implemented provisions tailored for students and teachers at the primary education level. These include organized workshops for school children and implemented university training courses for teachers (LaRue et al., 2018). These types of initiatives are designed both to educate children about genomics and to invoke critical thought about the benefits and drawbacks of genomic research. Additionally, these classical educational measures are supplemented by online platforms with resources for children and teachers.

#### Secondary Education Level

Genomic education at the secondary level is also an important measure toward increasing genomic literacy of the public since students not only use this knowledge in their later career but also often transfer their knowledge to their parents and other family members (Dressler et al., 2014). Students, who receive genomic education, are also more likely to participate in genomic research and request research results (Sabatello et al., 2019). Five projects included provisions for secondary education. Genome Quebec, a subsidiary of Genome Canada, offers an online platform for high school students and teachers, which doubles as an all-ages educational provision. Finland proposes incorporating genomic education into existing health education, thus providing students with sufficient resources to make informed decisions about their healthcare in the future.

#### Undergraduate Education

Good general genomic undergraduate education of science majors is necessary to recruit the next generation of genetic counselors, clinical and laboratory geneticists, and genetic nurses (Garber et al., 2016; Bennett et al., 2017; Whitley et al., 2020). Australia, Brazil, France, Slovenia, Switzerland, and the United Kingdom addressed undergraduate stakeholders. Brazil, Slovenia, Switzerland, and the United Kingdom organized educational events for students and healthcare professionals. Australia and France additionally introduced undergraduate and graduate programs, including transdisciplinary vocational programs, tailored for genomic professionals.

### Patients

Six projects included provisions for patients, focusing mostly on genomic education *via* workshops, online platforms, outreach programs, and informed consent provisions (Table 1; Figure 1).

Genomics England was one of the first projects to establish the Patient and Public Involvement Network, which tasked with the review of the educational resources and consent process. Patients were actively involved in the process of creating educational material and consent literature, including members of minority ethnic groups and youth. Their representatives were additionally involved through the Ethics Advisory Committee and other key institutions of the project in the management of the data access process, a crucial issue for study participants. Similarly, Australian Genomics put forward the Genomics in the Community Project in collaboration with Patient Advocacy Groups, focusing on analysis, curation, and preparation of educational material for patients and the public, on the topic of insurance and data privacy, an area where existing material was found to be insufficient.

Genome Canada and the Canadian Organisation for Rare Diseases are developing outreach programs aimed at patients with rare diseases to better understand the community of patients and tailor diagnostic and therapeutic approaches. Slovenian Genome Project will develop national guidelines for genomic medicine that will address genomic research and treatment, data management, interpretation of genetic results, biobanking, commercial genetic tests, and special interest groups. The project will develop national medico-ethical and legal frameworks for genomic medicine.

Australian Genomics designed the platform CTRL, a dynamic consent provision that enables the study participants to tailor and control the consent process, to receive news and study updates, and to contact the researchers. Rare Cancer Australia, a charity organization, offers a comprehensive online platform for patients with rare cancers with educational resources and tools for finding health services and clinical trials, a “Patient Treatment Found,” patient support services and support groups, and Radio Rare, a patient community-focused podcast.

### Professionals

All 16 projects included the education of professionals in genomic as their aim, reflecting its utmost importance (Table 1; Figure 1). As professional education includes several levels of formal education, the particular approaches to this challenge are discussed both under appropriate educational levels and as particular solutions implemented in the projects.

#### Graduate and Post-graduate Level

The majority of projects addressed the needs of graduate and postgraduate students with several projects establishing Master or PhD programs, focusing on building capacity and expertise in genomic medicine (Australia, France, Qatar, Slovenia, the United Kingdom, etc.). For example, Health Education England implemented a master’s degree in genomic medicine, targeting doctors and other healthcare professionals, with bioinformatics training utilizing the Genomic England dataset. Slovenia recently implemented a master PhD program for Genomic Counselors. Qatar implemented a master program in Genetic Counseling and a master and PhD program in Genomic Medicine. Australian Genomics established the Genomics Education Network of Australasia (GENA) to facilitate collaboration between providers of genomic education and implement new tools for genomic education, including a technical report of the overview of education programs for healthcare professionals involved in genomic medicine (McClaren et al., 2018). This report specifically identified the need to coordinate the implementation of new programs in response to the development of new technologies (McClaren et al., 2018). Other, broad higher-level educational resources include those of Cyprus that will establish the Centre of Excellence in Biobanking and Biomedical Research and Uruguay that implement tailored programs for researchers in collaboration with the University of Seoul.

#### Continuing Professional Development

We have identified five projects with tailored provisions for doctors and nurses that supplement existing specializations in genetics that are already part of formal education in countries such as Belgium (Hanquet, 2018). In addition to doctors and nurses, recent efforts have broadened the scope of professional genomic education to include non-medical healthcare workers that are nevertheless crucial for the development of genomics (Bennett et al., 2017). The projects specifically defining other target healthcare workers named diverse professionals, such as analysts, laboratory technicians, genetic counselors, researchers, pharmacists, data-scientists, bioinformatics engineers, system-administrators, government employees, industry staff, managers, etc. The professionals mentioned above can be loosely grouped under three categories – laboratory professionals, professionals in informatics, and others, such as research and development professionals, however, as shown by the approaches employed by national genomic projects these categories almost inevitably overlap to a large extent.

Common approaches to the education of working professionals consist of online and in-person educational events, such as workshops, seminars, lectures, summer schools, conferences, and training programs or initiatives. These approaches are similar to those included in the formal education of healthcare workers with the advantage of being more accessible to different professional profiles.

Specifically, Finland, Poland, and Saudi Arabia proposed strategies and programs to train existing healthcare professionals and develop new personnel in the field of genomics. Similarly, Australia’s National Health Genomics Policy Framework proposes strategies to increase the number of genetic professionals, increasing access to genetic professionals, and promote formal knowledge exchange with partnerships and networks (Australian Health Ministers’ Advisory Council, Australia, and Department of Health, 2017). Genomics Education England organizes an interactive course on the basics of genomic medicine in clinical practice aimed at nurses, general practitioners, other healthcare workers and scientists. Canada and Slovenia plan to organize educational events, including seminars, workshops, conferences, and courses on genomic data translation. Slovenian Genome project aims to create an online educational platform eSLOG. Finland plans to introduce guidelines, support tools, and databanks, to increase an utilization of genomic data for disease risk stratification. Similarly, Urugenome will implement a comprehensive training program for healthcare workers, particularly addressing the need for data analysis expertise, inclusion and report criteria for doctors, and ethical considerations. Finally, Brazil offers a biannual BIPmed workshop on technical development and current research, tailored for students, researchers, and managers from the public and private sector.

Six projects specifically describe provisions for bioinformatics engineers, solidifying the importance of data analysis and management for genetic testing and implementation of personalized medicine. The educational process for bioinformatics engineers is currently very diverse, which results in vastly different qualifications and carrier paths for professionals working in data analysis (Hanquet, 2018). France proposes implementing undergraduate and graduate transdisciplinary programs with recognized job titles such as biostatistics, data mining, and analysis. Their strategy proposes standardizing data analysis by providing modified training programs in bioinformatics and biostatistics and creating new job titles. Similarly, Japan’s Platform Program for Promotion of Genome Medicine aims to implement personalized medicine and disease prediction by educating engineers and researchers in bioinformatics and biostatistics, facilitating data management, data sharing, and genomic research of multifactorial diseases. Armenian Genome aims to promote education in bioinformatics and genomics by organizing workshops, seminars and providing research opportunities for researchers and students. New Zealand’s Genome Aotearoa implemented a comprehensive bioinformatics training program, which offers workshops and summer schools, and established a code repository within the GitHub online platform.

Illustrating the significance of competency in genomic medicine for other professionals involved in education, media and decision-making, Australia, Brazil, France, Poland, Switzerland, and the United Kingdom offer training programs and educational events for teachers, journalists, industry staff, insurance scientists, bioethicists, managers, and administrative staff.

An example to be followed, Genomics Aotearoa formulated the Guidelines for Genomic Research with Māori to equip researchers, ethics committee members and other professionals with a framework of ethical, social, and cultural considerations relevant to the Māori community of New Zealand.

### Limitations

The study faces several limitations, as previously described (Kovanda et al., 2021). Firstly, the authors gathered information available on the websites of the currently ongoing national genomic projects. Only the information available in the English language has been included, and we would like to recognize that our analysis may not reflect the full or final scope of the individual projects. The projects substantially differ in terms of their general scope and budgets. Additionally, the information on whether the impact of their educational measures will be evaluated was not available. We would like to emphasize that the primary focus of this review are the educational measures that are part of national genomic projects and recognize that many additional countries implement large scale national or even international initiatives and programs in genomic education outside the scope of their respective national genomic projects.

## Discussion

Genomic literacy empowers individuals to make informed decisions about their health, minimizes misconceptions about genomic testing and research, and leads to greater understanding and neutral perception of human diversity (Dressler et al., 2014).

In the context of national genomic projects, genomic education is primarily meant to increase project visibility and raise awareness about the implications of participation for the public and patients. The national genomic projects, discussed here, provide an opportunity to supplement the existing genomic education of professionals in their respective countries, facilitating the integration of personalized medicine into clinical practice (Wilcox et al., 2018). In medical terminology, national genomic projects’ educational measures are needed, but not themselves sufficient to achieve genomic literacy. Indeed, the scale of the effort needed implies each of the different existing measures (national, international, and project-type) can successfully contribute toward achieving genomic literacy.

The main challenges for genomic education of the public are limited financial resources, insufficient infrastructure, a lack of a unified approach, and the complexity of genomic medicine (Dressler et al., 2014).

Consequently, provisions for the public are often either broad or target a specific subgroup of the public (Figure 1). Indeed, in a 2014 study of genomic researchers and ELSI advisors, no consensus was reached on who should oversee the education of the public, what the target audience is and what topics should be presented (Dressler et al., 2014; Whitley et al., 2020). Providing opportunities for genomic education may not directly translate into an uptake of such opportunities or the use of the knowledge obtained, and it would be helpful to the field if educational measures could be evaluated in terms of their final impact.

Patients are the stakeholders most directly affected by genomics, and their adequate literacy on this topic is a fundamental step in the implementation of personalized medicine. Patient awareness regarding ELSI issues, especially data privacy, is a prerequisite of informed consent (Dressler et al., 2014). In addition to patients with rare diseases and cancer, genomic literacy is also important for patients with common non-communicable diseases, as low literacy puts these patients at a risk of not receiving personalized preventive care. On the other hand, an appropriate communication and interpretation of results represent unique challenges from the positions of the patient, parents/caretakers, and physicians/genetic councilors (Nakamura et al., 2017; von der Lippe et al., 2017; Stoller, 2018; Mboowa and Sserwadda, 2019).

The final and most complex group of stakeholders addressed by genomic projects is the healthcare and other professionals involved. In addition to doctors and nurses, professionals involved in genomics include laboratory analysts, informatics engineers, data scientists, administrators, legislators, ethics experts, and others, whose education presents additional challenges.

Due to the ever-increasing demand, substantial efforts to train genomic professionals exist independently of national genomic projects in many countries such as Belgium (Bennett et al., 2017; Hanquet, 2018). For an efficient implementation of genomic medicine, healthcare professionals both require and request educational resources that enable them to keep up to date with the current state-of-the-art in genomics (Ha et al., 2018; Hyland and Dasgupta, 2019; Cerovic et al., 2020). Several studies of European and United States physicians have illustrated that, although non-genetic physicians frequently see patients with rare diseases in their practice, they often lack formal education in genomic medicine, feel unprepared to order genetic tests, to interpret genomic data, and to effectively communicate the results to the patients (Rubanovich et al., 2018; Cerovic et al., 2020; Ramalle-Gómara et al., 2020). Similarly, nurses often lack the necessary genomic literacy. A 2018 survey of 18 countries showed that the integration of genomic education into nurse training was inconsistent and varied in scope (Calzone et al., 2018).

To facilitate an introduction to personalized medicine, medical students should be introduced to genomic medicine early on, with courses covering both preclinical and clinical medicine (Plunkett-Rondeau et al., 2015; Wilcox et al., 2018). Nationwide educational measures, such as the implementation of undergraduate and graduate programs, modification of school curriculum, and other large scale provisions, necessitate greater funding and collaboration between government institutions, universities, patient advocacy groups, etc. Several national genomic projects offer various solutions to supplement existing national educational structures (from primary to higher education), like establishing novel vocations or study programs or resorting to stand-alone solutions such as online resources and repositories for different professionals that can easily be tailored to specific groups.

Additionally, international collaboration contributes to achieving genomic literacy of professionals in genomics, by providing an overview of the state-of-the-art, access to new technology and databases, educational material, and other resources. Indeed, half of the national genomic projects included international provisions such as international conferences, courses for international students, collaboration in established international institutions and through open science initiatives, and organizing international projects. In these efforts, we have identified that the existing international initiatives, such as The Global Alliance for Genomics and Health (GA4GH), Elixir, ERN, Orphanet, etc., provide helpful educational resources and infrastructure for life science information (ELIXIR, n.d.; GA4GH, n.d.; Orphanet, n.d.; Public Health – European Commission, 2016). The adoption of existing international infrastructures (e.g., GA4GH, which provides policy frameworks and technical standards for secure and responsible data sharing), by national genomic projects, will hopefully translate to their national genomic education strategies in the future.

Finally, the educational provisions implemented by national genomic projects reflect the variability in the projects’ goals and unique national situations regarding genomics (Kovanda et al., 2021). The common approaches, presented in this mini-review, reflect the unique needs of different stakeholder groups, ethnic and cultural diversity, regulatory and legal genomic policies, and infrastructural capacities of the individual countries (Dressler et al., 2014; Mboowa and Sserwadda, 2019; Pastorino et al., 2019; Whitley et al., 2020). Due to the scope and the heterogeneity of genomics, as well as funding constraints, the most projects implemented cost-effective broad strategies, like educational events, online platforms, and community engagement projects, to educate the general public and patients, while professional training events, online platforms and tools, and an establishment of clinical guidelines and standards were used for educating of genomic professionals. Reflecting this variability, the predicted impact of increasing genomic literacy through genomic projects includes various factors contributing to developing personalized medicine, such as improved diagnostic capabilities, faster time to diagnosis of rare diseases, citizen and patient empowerment, greater visibility of genomic professions, and increased participation in genomic projects’ acquisition of normal and pathological genomic variability.

## Conclusion

In conclusion, we provide evidence of diverse educational activities across current national genomic projects, which reflect the differences in the goals of national genomic projects and specific national requirements. Examples of common approaches include workshops for healthcare workers, online information repositories for the general public and rare disease patients and families, and the development of guidelines, standards and national programs for implementation of genomic education into formal education. Hopefully, initial efforts made by national genomic projects will result in durable national solutions leading toward further implementation of personalized medicine in healthcare systems.

## Author Contributions

All authors listed have made a substantial, direct and intellectual contribution to the work, and approved it for publication.

## Conflict of Interest

The authors declare that the research was conducted in the absence of any commercial or financial relationships that could be construed as a potential conflict of interest.

## Publisher’s Note

All claims expressed in this article are solely those of the authors and do not necessarily represent those of their affiliated organizations, or those of the publisher, the editors and the reviewers. Any product that may be evaluated in this article, or claim that may be made by its manufacturer, is not guaranteed or endorsed by the publisher.
